# Interconnected Pathways of Alzheimer's Disease and Osteoporosis: A Review of Genetic, Hormonal, and Environmental Influences

**DOI:** 10.14336/AD.2024.1438

**Published:** 2026-02-10

**Authors:** Yu-Xin Li, Kun Liu, Qin Zeng, Wei-Hong Zeng, Jia-Li Li, Qian-Qian Zhang, Xuan Lu, Hong-Wen Deng, Li-Jun Tan

**Affiliations:** ^1^Laboratory of Molecular and Statistical Genetics, College of Life Sciences, Hunan Normal University, Changsha, Hunan 410081, China.; ^2^Tulane Center of Biomedical Informatics and Genomics, Deming Department of Medicine, Tulane University School of Medicine, New Orleans, LA 70112, USA.

**Keywords:** Alzheimer's disease, osteoporosis, hormones, genetic factors, environmental impact factors

## Abstract

Alzheimer's disease (AD) and osteoporosis (OP) are both age-related multifactorial degenerative diseases that share overlapping pathogenic mechanisms. While genetic factors contribute significantly to their onset and progression, environmental influences also play a crucial role. However, identifying the shared environmental factors and genetic architecture underlying the co-pathogenesis of AD and OP remains a significant challenge. This review highlights the common hormonal imbalances, environmental factors, genetic factors, and similar signaling pathways involved in both diseases. Furthermore, it explores the role of bone-secreted proteins and extracellular vesicles (EVs) in AD, as well as the effects of brain-derived proteins and EVs on bone homeostasis. By shedding light on the interconnected molecular and pathological mechanisms of AD and OP, this review aims to enhance our understanding of the underlying pathological molecular mechanisms of AD and OP, promote early diagnosis, and support the development of innovative therapeutic strategies for AD and OP patients.

## 1. Introduction

Alzheimer's disease (AD) is one of the most prevalent neurodegenerative disorders, with a high degree of heritability. Twin studies estimate the heritability of AD to range between 60% and 80% [1]. Clinically, AD is characterized by a progressive cognitive decline, behavioral abnormalities, and reduced ability to perform daily activities. Its histopathology hallmarks include the accumulation of extracellular amyloid beta (Aβ) plaques and intracellular neuronal (tau) tangles in the brain, which result in neuronal death, brain atrophy, and memory loss [2]. As the global population ages, the prevalence of AD continues to rise, with projections estimating 152.8 million cases worldwide by 2050 [3]. Despite extensive research, no curative treatments or effective therapies are currently available. The increasing incidence of AD poses a significant public health challenge, contributing to a substantial social and economic burden.

Osteoporosis (OP), the most common metabolic bone disease [4], is characterized by low bone mineral density (BMD), deterioration of the bone tissue microarchitecture [5], and reduced bone mass, which leads to an increased risk of fracture, particularly in elderly and post-menopausal females [6]. The clinical diagnosis and evaluation of OP primarily rely on the measurement of BMD. Similar to AD, BMD is a highly heritable trait, with heritability estimates ranging between 60% and 80% [7]. This indicates that genetic factors play a predominant role, while environmental factors also significantly influence bone density. Osteoporotic fractures have severe consequences, serving as a leading cause of mortality and disability in elderly patients, leading to a heavy burden on the healthcare system and society as a whole.

Genome-wide association studies (GWAS) have identified hundreds of susceptibility loci associated with OP and AD. However, the shared causal variants, genes, and molecular mechanisms underlying these two diseases remain largely unknown. To date, the genetic loci identified through GWAS account for only a fraction of the heritability of AD and OP, leaving a significant portion unexplained. While genetic factors play a critical role in the development of these conditions, environmental factors are equally important and contribute substantially to their onset and progression.

This review explores the interconnected pathways between AD and OP by examining genetic and environmental factors. It emphasizes the shared significance of these factors in the pathophysiology of AD and OP, focusing on their implications for prevention, early intervention, and the development of novel therapeutic strategies. Understanding these interconnections will provide valuable insights into the co-pathogenesis of AD and OP, paving the way for improved management of these age-related disorders.

Specifically, we conducted a systematic search of PubMed using the following keywords: “Alzheimer's disease”, “osteoporosis”, “brain”, “bone”, “osteocyte”, “estrogen”, “thyroid hormone”, “follicle-stimulating hormone”, “insulin”, “glucocorticoids ”, “melatonin”, “metal ions”, “sleep”, “diet ”, “exercise”, “smoking”, “inflammation”, “education level”, “air pollution”, “gut microflora”, “alcohol consumption”, “extracellular vesicles” This search focused on English-language studies up to November 2024, addressing genetic and environmental factors in AD and OP. All articles were screened for relevance based on their titles and abstracts. In addition, the reference lists of identified articles were reviewed to ensure the inclusion of pertinent studies not captured by the initial search. As a result, a total of 264 relevant articles in English were included in the review.

## 2. The complex relationship between AD and OP

Several studies suggest a potential association between AD and OP, but the evidence is inconsistent. Epidemiological research indicates that OP is a prevalent comorbidity in patients with AD, with individuals affected by AD frequently exhibiting OP and an increased incidence of hip fractures. Notably, femoral neck BMD has been associated with a twofold higher risk of developing AD and dementia in women [8]. Furthermore, clinical studies have demonstrated a strong correlation between bone metabolism biomarkers and early-stage AD [9]. Some studies have found that bone loss and OP are associated with the progression from mild cognitive impairment to AD [10]. A prospective cohort study reported that bone loss predicted subsequent cognitive decline in older women [11]. Recent studies involving large populations have also observed a significant bidirectional relationship between the rate of cognitive decline and bone loss, particularly in women [12].

Furthermore, reduced BMD has been linked to reduced left precuneus gray matter volume in memory-impaired older adults, suggesting that bone loss may influence cognition [13]. Conversely, a recent meta-analysis indicated a higher risk rate of cognitive impairment in patients with OP [14]. At the same time, a two-sample Mendelian randomization (MR) study found no bidirectional causal relationship between AD and OP [15]. These findings collectively indicate an association between AD and OP, but the supporting evidence remains somewhat conflicting and warrants further investigation.

## 3. Effects of hormones alteration on AD and OP

### 3.1 Estrogen

Estrogen, a type of steroid hormone, consists primarily of estrone, 17-beta estradiol, and estriol, with estradiol being the most biologically active form. Estrogen receptors are widely distributed throughout the body, including in the skeleton and brain [24]. Estrogen functions as an essential neurotrophic factor, playing a critical role in neurogenesis and maintaining the balance of brain function [25]. There is growing evidence that estrogen influences cognitive function through its interactions with various neurotransmitters, including brain-derived neurotrophic factor [26] (BDNF), acetylcholine [27], and dopamine [28]. Estrogen also exerts neuroprotective effects by inhibiting tau hyperphosphorylation and reducing Aβ deposition [29].

Furthermore, estrogen is essential for regulating glucose metabolism in the brain. Estrogen deficiency in postmenopausal women has been shown to elevate nitric oxide (NO) levels, leading to the production of s-nitrosylated complement factor C3 (SNO-C3), which activates microglia and ultimately contributes to cognitive decline in female patients [30]. The relationship between estrogen and AD has driven interest in hormone replacement therapy (HRT), which is associated with improved cognition in APOE4-positive women [31]. However, controversy exists regarding the safety and efficacy of HRT. For instance, a Finnish study found that long-term oral hormone (e.g., estrogen-only or estrogen-progestin combination tablets) therapy in postmenopausal women was associated with a slight increase in the risk of AD [32]. These contradictory findings underscore the need for more rigorous and comprehensive studies to clarify the role of HRT in AD prevention and management (Table 1).

**Table 1:** Correlation Study of AD and OP

Years	Study Population	Findings	Refs.
**1999**	8333 Elderly community-dwelling women enrolled	For every 1 SD decline in baseline hip BMD or calf bone BMD, the risk of cognitive decline increased by 32% (95% CI, 19-47%) or 33% (95% CI, 20-48%).	[16]
**2001**	4,304 elderly subjects	BMD in older adults was associated with verbal memory impairment.	[17]
**2003**	4462 women aged 70 and above	Women with more rapid hip bone loss are more likely to experience cognitive decline than women with lower rates of hip bone loss.	[11]
**2005**	987 subjects (610 female)	Reduced femoral neck BMD was associated with a twofold increased risk of AD in women.	[8]
**2010**	55 non-dementia participants and 63 early AD participants	Whole-body BMD was positively correlated with total hypothalamic volume in the early AD group (b0 = -1.93, b1 = 3.4, p = 0.007).	[18]
**2014**	946 men and women aged 60-75 years	Subjects with the lowest quartile BMD had a 2-fold increased risk of AD compared with controls.	[10]
**2018**	71 older adults with early AD and 69 non-dementia controls	Bone loss in AD may be related to neurodegenerative changes in the hypothalamus.	[19]
**2018**	29,983 OP patients and 29,983 controls	OP leads to a 1.2-fold increased risk of dementia in women and a 1.3-fold increased risk of dementia in men.	[20]
**2020**	42 male patients with early AD and 40 age-matched healthy older volunteers	Bone metabolism biomarkers and BMD are strongly associated with early AD patients.	[9]
**2020**	149 patients with memory disorders	Multiple regression analysis showed a significant positive correlation between BMD and rGMV in the left precuneus (adjusted *P* = 0.046 after SVC).	[13]
**2020**	1508 participants aged ≥60 years in the Bushehr Elderly Health (BEH) program in Iran	In women, bone loss and cognitive impairment may be risk factors for each other, but not in men.	[21]
**2021**	150 diagnosed AD patients, 150 sex- and age-matched healthy controls	86.7% of the AD patients showed low BMD.	[22]
**2021**	2361 participants ≥ 65 years of age	The rate of cognitive decline in women was significantly associated with bone loss, and the relationship was bidirectional.	[12]
**2021**	78,994 OP patients and 2015 controls	OP patients showed high risk of suffering AD (males HR=1.15; 95% CI = 1.04-1.28; P = 0.008; females HR=1.28; 95% CI = 1.22-1.34; P < 0.001).	[23]
**2023**	8 studies (136,222 subjects)	OP patients have a higher risk of cognitive impairment (OR=2.01, 95% CI=1.63-2.48, P<0.01).	[14]
**2023**	GWAS data for AD and BMD at different sites	There is no bi-directional causality between AD and OP.	[15]

AD, Alzheimer's disease; OP, osteoporosis; BMD, bone mineral density; rGMV, regional gray matter volume; SVC, small volume correction; HR, hazard ratios; OR, odds ratio; CI, confidence interval

In addition to its effects on the brain, estrogen plays a critical role in bone metabolism. A significant decline in estrogen levels in postmenopausal women leads to postmenopausal osteoporosis (PMO), a condition characterized by decreased bone mass and an elevated risk of fractures [33]. Estrogen deficiency accelerates bone resorption and impairs new bone formation, resulting in a marked reduction in BMD [34]. Estrogen promotes bone health by inducing osteoblast (OB) differentiation and reducing osteoclast (OC) activity. Moreover, it inhibits bone resorption by stimulating calcitonin production [35]. In the absence of adequate estrogen, calcitonin production decreases, leading to enhanced bone resorption and the development of OP.

Estrogen also interacts with the immune system, influencing the activity of various immune cells. For instance, during postmenopausal osteoporosis, estrogen deficiency activates B cells, which secrete higher levels of cytokines such as granulocyte colony-stimulating factor (G-CSF) and receptor activator of nuclear factor kappa-Β ligand (RANKL) [33]. To counteract these effects, selective estrogen receptor modulators, such as raloxifene, are commonly used for OP treatment and prevention. Ultra-low doses of estrogen (0.014 mg/day), approved by the FDA, have been shown to prevent BMD reduction in postmenopausal women, though its adverse effects require further investigation [36].

### 3.2 Thyroid hormone

Thyroid hormone (TH) exists in two primary active forms: tetraiodothyronine (T4) and triiodothyronine (T3) [37]. T4 is the predominant circulating form of TH, while T3 the biologically active form, is derived from the conversion of T4 within the body. TH is essential for normal growth and development, particularly in the brain and bones.

TH receptors are abundantly expressed in the brain [38]. Studies have demonstrated that thyroidectomy leads to hippocampus-dependent spatial learning and memory deficits in rats [39]. TH can also influence glucose metabolism and insulin signaling [37], both of which are critical for neural function and memory. Specifically, T3 signaling has been shown to inhibit amyloid-β precursor protein (APP) gene expression, thereby reducing Aβ formation through the modulation of histone H3 acetylation and methylation [40]. Consequently, low TH levels may accelerate AD progression by increasing APP expression, leading to elevated Aβ accumulation. Additionally, TH deficiency exacerbates AD pathology by influencing the immune response of microglia [41].

However, the relationship between TSH, TH, and cognitive performance remains controversial. For example, a population-based cohort study found no association between TSH or TH and the risk of AD, nor between TSH or T3 and brain atrophy [42]. In contrast, other studies have suggested that AD may develop in individuals with normal to high TSH levels [43]. While the exact mechanisms remain unclear, the potential influence of TH on the progression of AD cannot be overlooked. Understanding the alterations in TH metabolism within the AD brain may provide valuable insights into novel treatment approaches, including the use of TH replacement therapy.

In addition to its effects on the brain, TH plays a critical role in bone development and metabolism. TH binds to TH receptors on osteoblasts and osteoclasts in bone tissue, ensuring proper bone remodeling and bone metabolism. However, excessive TH reduces both the degree of mineralization of bone and the quantity of mineralized tissue, reflecting an acceleration in skeletal renewal [44]. Hyperthyroidism is a well-established trigger for high bone turnover, as excess TH enhances the activity of both osteoblasts and osteoclasts, ultimately leading to bone loss and increased fracture risk [45]. Conversely, hypothyroidism impairs bone remodeling by decreasing osteoblasts and osteoclasts activity, resulting in lower bone turnover, a prolonged bone remodeling cycle, and increased bone loss [4].

Understanding the dual effects of TH dysregulation on the brain and bones could pave the way for therapeutic strategies targeting both AD and OP. Future research should explore how optimizing TH levels through replacement therapy or other interventions could mitigate the pathology of these diseases.

### 3.3 Follicle-stimulating hormone

Follicle-stimulating hormone (FSH), a gonadotropin produced and secreted by the anterior pituitary gland, plays a critical role in regulating reproductive processes [46]. During menopause, women experience a sharp increase in FSH levels, often exceeding ten times their previous levels. A recent study published in Nature has revealed that elevated perimenopausal FSH levels are associated with the risk of AD, as higher FSH levels accelerate the deposition of Aβ peptides and tau protein in the brain [47]. This finding offers important insights into why women are more susceptible to developing AD compared to men.

In addition to its effects on the brain, FSH is increasingly recognized as a regulator of bone resorption, acting independently of estrogen [48]. Notably, selective FSH blockade stimulates new bone formation [47]. Clinical evidence suggests that serum FSH levels negatively correlate with BMD and positively correlate with bone turnover markers [49]. Elevated FSH levels are strongly linked to menopausal bone loss. Experimental studies, both in vitro and in vivo, have demonstrated that FSH directly or indirectly impacts bone health [50]. FSH binds to its receptor (FSHR) on osteoclasts, promoting osteoclastogenesis and enhancing bone resorption [51]. Additionally, FSH stimulates the production of tumor necrosis factor-alpha (TNFα), which stimulates osteoclast precursor expansion and osteoblast differentiation [52]. The dual role of FSH in regulating bone mass and modulating cognitive function underscores its significance in the interconnected pathways of AD and OP. While these findings highlight the importance of FSH and its receptor (FSHR) in bone and brain health, there remains uncertainty regarding whether elevated FSH levels or decreased estrogen levels are the primary drivers of postmenopausal osteoporosis. Further research is required to elucidate the relative contributions of these hormonal changes to the pathogenesis of both AD and OP.

### 3.4 Insulin

Insulin, a peptide hormone secreted by the pancreas, plays a critical role in regulating glucose metabolism in peripheral tissues [53]. Insulin receptors are abundantly expressed in key brain regions, including the hippocampus, mesencephalon, and temporal lobe, which are particularly vulnerable to neurodegeneration [54]. This suggests that altered glucose metabolism in the brain may contribute to the pathogenesis of AD [55]. Insulin has a role in maintaining protein homeostasis by influencing the clearance of Aβ peptide and the phosphorylation of tau, two hallmarks of AD [56]. A study showed that peripheral insulin resistance in AD patients positively correlates with increased brain Aβ deposition, particularly in frontal and temporal lobe regions [57]. There is growing evidence that insulin dysregulation exacerbates Aβ deposition and hyperphosphorylation of Tau proteins, significantly increasing the risk of AD.

Insulin dysregulation also impacts bone health. Abnormal glucose and lipid metabolism associated with insulin dysfunction in the AD brain can lead to secondary bone deterioration. Glucose is an essential nutrient for osteoblasts [58], and bone relies heavily on glucose metabolism. Insulin acts as a crucial regulator of bone growth, influencing calcium resorption and bone mineralization. Insulin signaling is vital for osteoblast differentiation and proliferation, as it promotes Runx2 expression, a key transcription factor that facilitates bone formation [59]. Additionally, insulin stimulates osteoblast differentiation, enhances intracellular amino acid accumulation, and supports the synthesis and secretion of bone collagen and bone matrix while promoting the production of osteocalcin [60].

On the other hand, insulin also modulates osteoclast activity. Elevated insulin levels affect osteoclast recruitment and differentiation by impairing the RANKL signaling pathway [61]. Conversely, insufficient insulin secretion compromises osteoblast-mediated bone regeneration while increasing osteoclast-driven bone resorption. This imbalance leads to a higher rate of bone resorption compared to bone formation, which ultimately accelerates the development of OP.

The intricate interplay between insulin, glucose metabolism, and bone homeostasis underscores the significance of insulin dysregulation in the interconnected pathways of AD and OP. Further research is needed to understand these mechanisms better and explore insulin-targeted interventions for the prevention and management of both AD and OP.

### 3.5 Glucocorticosteroids

Glucocorticosteroids (GC) are steroid hormones capable of freely crossing the blood-brain barrier, where they bind to a high-affinity mineralocorticoid receptor (MR) and low-affinity glucocorticoid receptor (GR). Numerous animal studies have demonstrated that elevated GC levels contribute to the pathophysiology of AD through several mechanisms, including increased Aβ deposition and tau protein hyperphosphorylation, chronic inflammation, and impairment of synaptic plasticity. Transgenic mouse experiments have provided further insights into the role of GCs in AD, revealing that elevated GC levels exacerbate tau hyperphosphorylation and amyloid accumulation [62]. Additionally, abnormally elevated GC levels affect the structure and function of the hippocampal synapses [63], which can result in cognitive and memory impairment. Elevated GC levels, coupled with microglia abnormalities, also induce chronic brain inflammation [64] and neurotoxicity, ultimately contributing to AD progression.

Glucocorticoid-induced osteoporosis (GIOP) is the most common form of secondary osteoporosis [65]. GCs directly influence the function of osteoblasts, osteoclasts, and osteocytes [66]. Specifically, GCs inhibit osteoblast differentiation and maturation [67], leading to decreased bone formation. Moreover, GCs directly prolong osteoclast longevity [68], resulting in decreased BMD. They also increase the ratio of RANKL to osteoprotegerin, promoting osteoclast differentiation and maturation, which further accelerates bone resorption. [69]. Paradoxically, long-term GC therapy inhibits osteoclasts, thereby impairing bone remodeling [70]. Beyond their direct effects, GCs reduce intestinal absorption of calcium [71], suppress estrogen and testosterone synthesis [65], and decrease muscle mass, all indirectly contributing to bone loss.

While GCs are essential for the treatment of numerous conditions due to their potent anti-inflammatory and immunosuppressive effects, their overuse poses significant risks for OP. Thus, careful control of GC dosage is critical to minimizing adverse effects. Bisphosphonates, such as alendronate [76]), have demonstrated efficacy in treating GIOP, postmenopausal OP, and male OP. Notably, recent studies have found that alendronate may also inhibit Aβ deposition and memory loss in AD transgenic mice [77]. A population-based analysis found that OP patients treated with bisphosphonates exhibited a significantly lower risk of developing dementia compared to untreated patients [78].

### 3.6 Melatonin

Melatonin is an amine hormone synthesized by the pineal gland, with its production initiated from the amino acid tryptophan [72]. Melatonin, an essential hormone that affects the As a critical regulator of circadian rhythm, melatonin has been shown to exert significant neuroprotective effects, including attenuation of Aβ-induced intracerebral neurotoxicity [73] and may prevent or slow the progression of AD. Experimental evidence demonstrates that melatonin can improve cognitive deficits in AD mouse models by promoting mitochondrial autophagy [74]. Increasing studies highlight the potential of melatonin in AD treatment. For instance, scientists discovered that melatonin and its metabolites may preserve memory by protecting against cognitive decline in mice and humans [75]. Mechanistic studies further revealed that melatonin mediates the degradation of death-associated protein kinase 1 (DAPK1) and enhances the activity of Pin1, a prolyl isomerase with a protective role against tau hyperphosphorylation and tau-related pathology in AD [76]. These findings position melatonin as a promising candidate for AD treatment and as a potential anti-aging agent, providing new options for AD therapy.

Melatonin also plays an integral role in maintaining bone metabolism by regulating the balance between bone resorption and bone formation. As early as 1999, ROTH et al. [77] reported that melatonin increases the expression of bone sialoprotein (BSP) as well as several key bone marker proteins (e.g., ALP), thereby promoting osteoblast differentiation. Recent studies have demonstrated that melatonin counteracts glucocorticoid-induced inhibition of osteoblast differentiation via the PI3K/AKT and bone morphogenetic protein/Smad signaling pathways [78]. Animal studies corroborate these findings, showing that melatonin facilitates osteoblast differentiation while suppressing osteoclast formation through mechanisms such as reducing oxidative stress and inhibiting peroxisome proliferator-activated receptor gamma (PPARγ) [79]. Moreover, melatonin inhibits bone resorption by downregulating RANKL-mediated osteoclast activation [80].

Emerging evidence suggests that oxidative stress is a major pathogenic mechanism factor in OP. Melatonin enhances cellular antioxidant capacity by activating multiple signaling pathways, maintaining mitochondrial stability [81], and inhibiting apoptosis. These processes mitigate cytotoxic damage, promote cellular osteogenic differentiation and mineralization [82], and suppress osteoclast differentiation [83]. Recently, mutations in the melatonin receptor 1A (MTNR1A) have been identified as a genetic cause of idiopathic osteoporosis (IOP) [84], providing further evidence for the repurposing of melatonin as a potential therapy for OP. Collectively, these findings highlight the dual roles of melatonin in neuroprotection and bone health, underscoring its potential as a novel therapeutic strategy for AD and OP (Table 2).

**Table 2:** The effects of different hormones on AD and OP.

Hormones	Association mechanism	Refs.
**Estrogen**	Affects cognitive function; Promote bone formation and inhibit bone resorption	[29, 35, 85]
**Thyroid hormone (TH)**	Affects glucose metabolism and APP expression; Affects bone resorption and bone formation	[4, 37, 40, 45]
**Follicle-stimulating hormone (FSH)**	Affects the deposition of Aβ peptide and tau protein; Increases osteoclastogenesis and resorption	[47, 48]
**Insulin**	Affects the clearance of Aβ peptide and phosphorylation of tau; Affects calcium absorption and bone formation	[56, 59]
**Glucocorticoids (GC)**	Affects Aβ deposition, tau protein hyperphosphorylation, and brain inflammation; Affects bone formation	[62, 68, 86]
**Melatonin**	Affects sleep and cognitive function; Promote bone formation and inhibit bone resorption	[74, 78, 79]

AD, Alzheimer's disease; OP, osteoporosis; Aβ, amyloid beta

## 4. The impact of environmental factors on AD and OP

### 4.1 Metal ion

The appropriate balance of metal elements, such as zinc, iron, and copper, is essential for regulating human growth, brain development, and metabolism. Increasing evidence suggests that abnormalities in metal ion metabolism in the brain are a significant risk factor in the pathogenesis of AD [87]. Metal ions play a critical role in the APP hydrolysis process [88]. Research has revealed that the balance of metal ions in AD patients is disrupted, with iron, zinc, and copper levels in the brain being 3-7 times higher than those in healthy individuals [89]. Notably, iron levels in the brain increase with age, and iron accumulation is directly related to the number of Aβ plaques, particularly in plaques and microglia in the frontal cortex of AD patients [90]. A recent study has identified that the multitarget iron chelating and propargylamine drug M30 has neuroprotective effects in AD models [91]. Similar to iron, copper is involved in cellular oxidative stress and Aβ production [92]. These findings suggest that the dysregulation of metal ion homeostasis significantly impacts APP processing and A β generation, playing an important role in the pathological changes of AD. Therefore, metal ions may be a plausible therapeutic target for AD, with potential treatments focusing on modulating metal ion levels to restore balance.

In recent years, the role of metallic elements in the pathogenesis of OP has also attracted considerable attention. Metallic trace elements, such as manganese, iron, zinc, and copper, are integral components of various catalytic enzymes or coenzymes, regulating bone formation and bone turnover processes. Imbalances in these elements can disrupt bone mineral formation and bone matrix synthesis, ultimately contributing to the development of OP [93]. For instance, iron overload and iron deficiency have been linked to increased risk of OP. Iron overload inhibits osteoblast activity [94], suppressing bone formation and causing bone damage. In contrast, mild iron deficiency may stimulate osteoblast activity, whereas severe iron deficiency inhibits osteoblast function [95], leading to an imbalance in bone metabolism and a heightened risk of OP.

### 4.2 Sleep

Sleep is a fundamental physiological activity intricately related to circadian rhythms and is considered an essential process for the brain to repair and store information. Normal sleep facilitates the clearance of metabolites and harmful proteins, including Aβ, from the brain. Sleep disorders are a risk factor for AD, with affected individuals experiencing a more significant accumulation of neurotoxic substances due to impaired metabolite clearance [96]. Notably, there appears to be a bidirectional relationship between sleep disorders and AD pathology. Sleep disorders promote the accumulation of Aβ and tau proteins, while increased Aβ and tau aggregation may, in turn, exacerbate the progression of sleep disorders [97]. Chronic sleep deprivation plays a key role in regulating microglia inflammatory and metabolic responses and contributes to increased Aβ plaque deposition in the brain. Early-life chronic sleep deprivation has been linked to AD-related impairments in glycolysis, disrupting metabolic balance, resulting in aberrant protein deposition, and ultimately impairing cognitive function [98]. These findings underscore the importance of adequate and high-quality sleep in reducing Aβ accumulation and mitigating AD risk. Addressing sleep disorders is crucial for AD prevention and treatment. Slow wave activity (SWA) during non-rapid eye movement sleep (NREM) has shown the potential to mitigate memory loss in individuals with significant AD pathology [99].

Sleep disturbances have also been implicated as risk factors for OP. Experimental studies in rats [100] and healthy men [101] have shown that sleep and circadian rhythm disturbances impair bone formation. Both sleep deprivation and excessive sleep are associated with decreased bone density and an elevated risk of developing OP. Animal experiments have found that chronic sleep deprivation during growth phases negatively affects skeletal development, specifically by inhibiting bone formation [102]. Furthermore, several studies have identified chronic sleep disorders as significant risk factors for OP [103]. Poor sleep quality and excessive or insufficient sleep adversely impact bone density, underscoring the necessity of maintaining an optimal sleep duration for bone health.

### 4.3 Lifestyle

#### 4.3.1 Diet

The relationship between diet, bone health, and brain health has received a lot of attention in recent years. Unhealthy dietary patterns, including high-fat and high-salt diets, have been shown to affect various physiological processes adversely. Long-term consumption of a high-fat diet (HFD) not only increases the risk of obesity but also induces chronic brain inflammation, which can impair cognitive function. High-fat diets are directly implicated in the pathogenesis of AD through mechanisms such as enhanced Aβ accumulation, tau protein hyper-phosphorylation, and exacerbation of neuroinflammation [104]. Moreover, HFD consumption has been shown to alter gut microbial composition [105], triggering systemic inflammation. Inflammation, an important regulator of bone remodeling, can impede bone formation and increase the risk of OP [106]. Additionally, HFDs reduce calcium absorption in the intestines and kidneys, leading to diminished bone minerals (calcium and phosphorus) [107] and subsequent bone loss, thereby increasing susceptibility to OP.

A high-salt diet (HSD) poses similar risks to both cognitive and skeletal health. HSDs have been identified as risk factors for dementia, as they promote tau protein phosphorylation in the brain, leading to cognitive impairment [108]. Elevated sodium intake has been shown to increase blood levels of interleukin 17 (IL-17), which reduces nitric oxide (NO) bioavailability, resulting in imbalanced vasoregulation [109]. This imbalance restricts the oxygen and glucose supply to brain regions critical for cognitive function. Additionally, NO depletion exacerbates hyperphosphorylation of tau proteins [110], further contributing to cognitive deficits in mice.

Excessive salt intake also negatively affects bone health. In addition, HSDs increase calcium loss from the human endoskeleton by promoting urinary calcium excretion, which can accelerate bone demineralization and heighten the risk of OP. [111]. A recent study based on the Korean population data revealed a negative correlation between urinary sodium excretion and bone health, indicating that high salt intake is a significant risk factor for OP [112].

The Mediterranean diet, characterized by a high intake of vegetables, fruits, legumes, fish, and olive oil, promotes brain and bone health [113, 114]. Studies have linked this diet to increased lumbar spine BMD in postmenopausal women [115] and a reduced risk of cognitive decline and AD [116, 117].

#### 4.3.2 Exercise

An accessible and efficient method to slow brain aging is to increase physical activity. Studies have shown that regular exercise improves brain health and is associated with increased brain volume [118]. Recent research indicates that exercise can alleviate the pathological features of AD and improve cognitive dysfunction by modulating the morphology and function of astrocytes. Clinical studies have demonstrated that regular physical activity significantly improves mental health and cognitive performance in healthy older adults and AD patients. For instance, a prospective cohort study conducted on the Japanese general population revealed that higher participation in daily physical activity was associated with nearly a 50% reduction in AD risk [119]. Moreover, recent findings suggest that moderate physical activity (MPA) is particularly effective in lowering the risk of developing AD [120]. Additionally, long-term exercise has been shown to enhance sleep quality, which indirectly contributes to improved brain health.

Regular exercise also plays a pivotal role in maintaining bone health during aging and is widely recognized as an effective intervention for promoting bone formation in OP patients. Mechanically induced forces from physical activity are essential for maintaining bone homeostasis, as the absence of exercise deteriorates bone strength and structure and accelerates bone loss [121]. Exercise increases lumbar spine and femur BMD in menopausal women and older adults [122, 123].

### 4.4 Smoking

The dangers of smoking extend far beyond its well-documented effects on lung health, posing a serious threat to brain health and increasing the risk of AD. Research has shown that middle-aged smokers are more likely to experience memory loss, cognitive impairment, and AD compared to non-smokers [124]. A study reported that smoking is associated with accelerated degradation of gray and white matter [125]. Furthermore, smoking has been shown to shrink the brain and accelerate brain aging; notably, this reduction in brain volume appears to be irreversible [125]. Smoking also has a profound impact on the cerebral cortex, which is significantly thinner in smokers compared to ex-smokers and non-smokers [126]. This cortical thinning is a key marker of neurodegeneration and may signal an increased dementia risk 5-10 years before the onset of clinical symptoms [127].

In addition to its detrimental effects on brain health, smoking compromises bone health significantly by reducing bone strength and BMD. A previous study found that active smokers have lower BMD compared to non-smokers, putting them at a higher risk for OP and fracture [128]. GWAS analysis reveals a significant genetic correlation between smoking status and BMD, indicating that smoking behavior and BMD are influenced by shared genetic factors [129]. Cigarette smoke contains a variety of toxic and hazardous substances such as nicotine, heavy metals (arsenic, cadmium, and lead), and tar, which collectively harm the skeletal system and reduce BMD. Cadmium, in particular, is a major contributor to smoking-induced bone damage, as the element accumulates in the body over time, with bone being a primary target of cadmium toxicity [130].

Nicotine, another key component of cigarette smoke, exhibits dose-dependent effects on bone metabolism. Low nicotine concentrations promote osteoblast-like cell proliferation and the expression of genes involved in bone metabolism, while high nicotine concentrations exert the opposite effect [131]. Additionally, polycyclic aromatic hydrocarbons (PAH), such as benzo [a]pyrene (BaP), a carcinogenic compound found in cigarette smoke, have been shown to inhibit osteoclast differentiation and bone resorption through crosstalk between RANKL and NF-κB [132]. Smoking has also been linked to an increased risk of insulin resistance, which may further contribute to compromised bone health [133]. Smoking also disrupts hormonal balance, reducing estrogen production and metabolism [134], thereby diminishing the protective effects of estrogen on bone health in premenopausal women.

Notably, passive smoking has been shown to produce effects comparable to those of active smoking, suggesting that both are significant risk factors for OP. The Bone Health and Osteoporosis Foundation (BHOF) strongly advocates for smoking cessation as a preventive measure against OP.

### 4.5 Inflammation

Inflammation is the body's immune response to injury, infection, or other stimuli. The brain is particularly vulnerable to inflammation, and neuroinflammation is prominently observed in pathologically affected regions in AD [135]. While the exact etiology of AD remains elusive, neuroinflammation is increasingly recognized as a crucial contributor to its development and progression. Microglia, the primary immune cells in the central nervous system (CNS), play a central role in neuroinflammation. Upon activation, microglia release proinflammatory cytokines, such as TNF, which have been shown to induce tau accumulation in neurons [136]. Persistent neuroimmune inflammation is now considered the third hallmark of AD, alongside Aβ plaques and neurofibrillary tangles (NFTs) [137].

The role of inflammation in AD is complex, as it exhibits both protective and detrimental effects depending on the stage of the disease. While attenuation of pro-inflammatory phagocytosis during the early stage of Aβ plaque accumulation can have harmful effects, chronic activation of pro-inflammatory signaling exacerbates tau pathology, synaptic loss, and neuronal damage [138].In addition, chronic inflammation is a key factor in blood-brain barrier (BBB) dysfunction, which further impairs Aβ metabolism and accelerates its deposition [139, 140]. These findings underscore the central role of neuroinflammation not only as a response to the pathological process of AD but also as a driving force in the progression of AD. Consequently, numerous therapeutic strategies targeting neuroinflammation have been developed and are currently undergoing clinical trials.

Inflammation also exerts a profound impact on bone metabolism, disrupting the delicate balance between bone resorption and bone formation [141]. Chronic inflammation promotes pathological bone loss by increasing osteoclastic activity while inhibiting osteoblastic function [142]. Inflammatory states are characterized by elevated levels of pro-inflammatory factors such as TNF-α and interleukin-6 (IL-6), which are major regulators of dysregulated bone remodeling [143]. TNF-α activates the NF-κB pathway in osteocytes, upregulates RANKL, and stimulates osteoclastogenesis and bone resorption [144]. Simultaneously, TNF-α suppresses osteoblast activity, further reducing bone formation and increasing the risk of OP. IL6 also promotes osteoclastogenesis [145] and is an important biomarker for postmenopausal osteoporosis and a predictor of osteoporotic fractures [146].

The interplay between inflammation, AD, and OP highlights a shared pathogenic mechanism that involves dysregulated immune responses. Chronic inflammation not only accelerates the progression of AD but also contributes to abnormal bone metabolism, emphasizing the need for therapeutic interventions that target inflammatory pathways to mitigate the effects of these interconnected diseases.

### 4.6 Educational level

Higher levels of education are widely believed to enhance an individual's cognitive reserve, defined as the brain's capacity to maintain cognitive function as long as possible under any damaging conditions [147]. A study by Larsson [148] et al. identified a significant association between higher educational attainment and a reduced risk of AD, emphasizing the protective role of education in cognitive health. Recent research also demonstrates a significant correlation between years of education and a reduced risk of AD [149]. However, once AD symptoms manifest, a study has also noted that individuals with high cognitive reserve showed a faster rate of cognitive decline compared to those with lower cognitive reserve [150]. This accelerated decline may reflect the ability of cognitive reserve to mask early symptoms, delaying diagnosis until the pathology has advanced significantly. These findings highlight the need to increase access to education and promote cognitive activities that stimulate the population across all ages as a preventive strategy for AD.

Educational attainment also plays a crucial role in the prevention and management of OP. Lower levels of education have been identified as an independent risk factor for OP [151]. Conversely, higher levels of education are positively associated with OP prevention, as they are linked to broader perspectives, greater access to knowledge, and more proactive attitudes toward bone health [152]. Educated individuals are more likely to engage in health-promoting behaviors, access high-quality healthcare, and exhibit higher health literacy [153]. These advantages contribute to a healthier lifestyle, including balanced nutrition, regular physical activity, and reduced exposure to environmental toxins, which collectively lower the risk of developing AD and OP.

The interplay between education, AD, and OP underscores the importance of societal efforts to enhance educational opportunities and health literacy. By promoting lifelong learning and awareness, it may be possible to mitigate the risks of these interconnected diseases through improved cognitive reserve and healthier lifestyles.

### 4.7 Air pollution

Air pollution is the fourth leading cause of illness and mortality globally [154]. Long-term exposure to air pollution, particularly fine particulate matter (PM2.5), has been implicated in the pathophysiology of AD [155]. In 2018, The Lancet Pollution Commission identified a strong association between atmospheric particulate matter and the development of dementia in the elderly population [156]. PM2.5 has been shown to alter the permeability of the BBB and thus enter the brain. In addition, PM2.5 can also enter the brain by traveling along the olfactory nerve via the nasal mucosa. Emerging evidence suggests that interactions between PM2.5 and genetic factors, such as APOE, may hasten brain aging, offering novel insights into the pathogenesis of AD [157]. A recent study analyzed postmortem brain tissue from 224 individuals and revealed that those exposed to elevated PM2.5 levels for at least one year before death exhibited higher levels of amyloid plaques [158]. Remarkably, even PM2.5 concentrations below the air quality standards set by the US EPA, the UK, and the EU were found to significantly increase dementia risk [159]. These findings indicate that prolonged exposure to low concentrations of PM2.5 is sufficient to elevate the risk of developing AD, highlighting the urgent need for stricter air quality regulations and public health interventions.

In addition to its impact on cognitive health, poor air quality is emerging as a significant risk factor for bone health. A prospective observational study explored the relationship between air pollution and bone health and identified nitrogen oxides as key contributors to bone loss, particularly in the lumbar spine, the most susceptible site. [160]. Recent experimental evidence from in vitro and in vivo models further supports these findings, showing that PM2.5 exposure may increase the risk of OP by promoting osteoclastogenesis through the TNF-α-mediated Traf6/c-Fos pathway [161].

The dual impact of air pollution on both AD and OP underscores its far-reaching consequences on public health. Efforts to mitigate air pollution could significantly reduce the prevalence of these interconnected diseases.

### 4.8 Gut microbiota

The gut microbiota refers to the diverse community of microorganisms residing in the intestinal tract, which interacts with the CNS in a bidirectional manner, forming the gut-brain axis. This interaction is primarily mediated through the production of microbial metabolites, as well as the modulation of the nervous, immune, and endocrine systems [162].

Recent studies have highlighted the significant role of gut microbiota in the pathogenesis of AD. Gut microbiota-derived metabolites can enhance inflammatory response in the CNS, leading to pathologic microglial function, increased neurotoxicity, and impaired amyloid clearance, collectively heightening AD risk [163]. A recent amplicon sequencing technology study identified the gut as a potential source of Aβ production. Gut microbiota can upregulate gut-derived Aβ, contributing to the pathogenesis of AD [164]. Studies of the gut microbiota in AD patients have revealed an increased abundance of pro-inflammatory microorganisms and a reduced population of beneficial metabolite-producing microorganisms, establishing a causal relationship between AD and gut microbiota dysbiosis [165].

The intimate relationship between intestinal flora and AD development is now well-documented, with gut microbiota and their metabolites playing pivotal roles in neuroinflammation and amyloid pathology. These findings suggest that interventions to regulate intestinal flora may represent a novel strategy for combating AD by mitigating neuroinflammation via the brain-gut axis. Novel therapies targeting the brain-gut axis show promise. For example, chiral Au nanoparticles and sodium Oligomannate (GV-971) have been shown to modulate gut flora, reduce neuroinflammation, and improve cognitive function in AD models [172-174].

The gut microbiota also plays a crucial role in regulating bone metabolism by modulating intestinal function, immune system, and endocrine system [166, 167]. Healthy gut microbiota can reduce intestinal inflammation and improve bone metabolic function [168]. Recent studies have revealed a possible mechanism linking gut bacteria to bone health. For example, Bacteroides vulgaris has been shown to negatively impact bone structure in mice by inhibiting the production of valeric acid, thereby promoting bone absorption [169]. In contrast, short-chain fatty acids (SCFAs (, produced by the gut microbiota, exhibit anti-inflammatory effects by inhibiting the activation of nuclear factor kappa-light-chain-enhancer of activated B cells (NF-κB ( activation [170]. SCFAs also regulate osteocyte metabolism and bone mass by regulating osteoclast differentiation and inhibiting excessive bone resorption without impairing bone formation [171].

Furthermore, the gut microbiota promotes insulin-like growth factor 1 (IGF-1) production via SCFA-mediated pathway. IGF-1 stimulates osteoblast, osteoclast, and chondrocyte differentiation, which are vital for bone remodeling [172]. The gut microbiota also synthesizes key vitamins, such as vitamin B [173] and vitamin K [174], which are crucial for maintaining bone health. In addition, the gut microbiota influences the nervous system by regulating the synthesis of hormones and neurotransmitters such as serotonin (5-HT) [175, 176]. Serotonin signaling, in particular, plays a crucial role in bone development and maintenance [177].

The evidence suggests that the gut microbiota may form an "axis" connecting the gut to bone metabolism, influencing bone health through intricate pathways involving inflammation, endocrine signaling, and vitamin synthesis. Therefore, maintaining the homeostatic balance of gut microbiota holds significant potential for promoting cognitive and skeletal health.

### 4.9 Alcohol consumption

Alcohol and its metabolite acetaldehyde exert direct neurotoxic effects, causing permanent structural and functional damage to the brain [178]. Evidence suggests that alcohol consumption accelerates the pathological cascade of AD, particularly in its early stages. Even moderate alcohol consumption has been associated with accelerated brain atrophy and increased amyloid plaque deposition [179]. Animal studies have shown that chronic alcohol consumption leads to neuronal tau protein phosphorylation in the hippocampus, resulting in impaired memory and cognitive deficits [180]. Furthermore, a large-scale study analyzing data from over 36,000 adults in the UK Biobank found that alcohol consumption is associated with reduced brain volume. Strikingly, even light to moderate alcohol consumption was found to have detrimental effects on brain health, with an average of one beer per day associated with brain aging comparable to two additional years of natural aging [181]. These findings challenge earlier perceptions of moderate alcohol use being relatively harmless, highlighting that even low levels of alcohol consumption can significantly compromise brain health and contribute to AD risk.

In addition to its effects on cognitive health, chronic alcohol consumption is a major risk factor for alcohol-induced osteoporosis (AOP), a secondary form of osteoporosis. Research demonstrated that long-term alcohol consumption leads to heightened secretion of TNF-a from peripheral monocytes and tissue-resident macrophages in rhesus monkeys, accompanied by upregulated expression of genes linked to oxidative stress and inflammation/cellular activation [182]. Alcohol disrupts bone cell activity by inhibiting the proliferation and differentiation of bone marrow mesenchymal stem cells (BMSCs) into osteoblasts [183]. Chronic and excessive alcohol consumption interferes with bone remodeling, leading to significant bone loss [184, 185]. Recent studies have further shown that excessive alcohol consumption decreases trabecular bone area ratio and osteoblast numbers, while increasing osteoclast numbers, resulting in an overall imbalance in bone remodeling [186]. Alcohol consumption alters gut microbiota composition, causing intestinal calcium malabsorption, elevated parathyroid hormone (PTH) levels, reduced production of the active form of vitamin D, and disrupted the production of sex hormones, all of which exacerbate bone loss [187].

Interestingly, some research suggests that the detrimental effects of alcohol on the bone are partially reversible. Abstinence from alcohol has been associated with increased BMD; however, it remains unclear whether BMD will return to normal levels in long-term abstainers [184].

**Figure 1.**
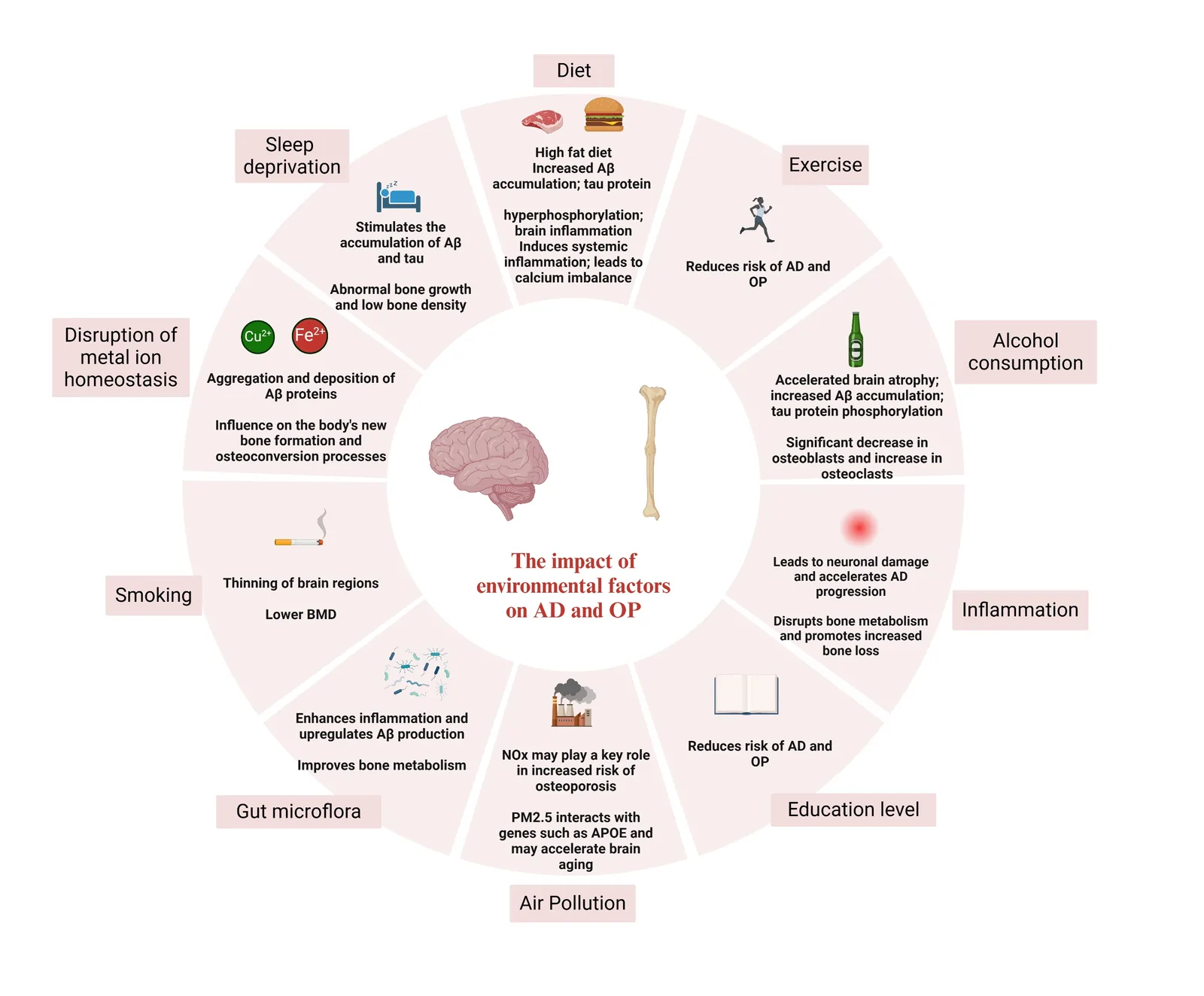
**The impact of environmental factors on AD and OP**. AD, Alzheimer's disease; OP, osteoporosis; Aβ, amyloid beta; BMD, bone mineral density (Created with BioRender.com)

The dual role of alcohol in exacerbating both AD pathology and OP highlights the necessity of addressing alcohol consumption as a modifiable risk factor for these interconnected diseases. Reducing alcohol intake or achieving abstinence may offer significant benefits for both cognitive and skeletal health (Fig. 1).

## 5. Potential common genetic factors

### 5.1 TREM2

Trigger receptor expressed on myeloid cells 2 (TREM2) is an innate immune receptor expressed on the cell surface of myeloid cells, including preosteoclasts, microglia, dendritic cells, and macrophages. TREM2 has emerged as a key genetic risk factor for AD and plays a pivotal role in both neuroinflammation and bone metabolism [188].

In the CNS, TREM2 is critical for maintaining immune homeostasis and preventing neurodegeneration. It achieves this by promoting phagocytosis, regulating microglia proliferation and survival, controlling cytokine release, and mediating accumulation around Aβ plaques [189, 190]. In addition, TREM2 activation in microglia also attenuates harmful inflammatory responses, which are central to AD pathology [191]. These functions make TREM2 an essential modulator of the neuroimmune response in AD, linking its activity to the regulation of Aβ pathology and neuroprotection.

Beyond its role in CNS, TREM2 is also a key regulator of bone metabolism, functioning as a crucial factor in osteoclast differentiation and activity. TREM2 is implicated in the activation of the classical Wnt/β-catenin pathway, which serves as a critical molecular link between bone and brain health [192]. Experiments evidence from mouse models have shown that TREM2 and β-catenin jointly regulate bone resorption by modulating the rate of osteoclastogenesis [193]. Furthermore, TREM2 blockade prevents functional resorption of OCs and regulates OC migration [194]. TREM2 is emerging as a potential therapeutic target for AD and OP. Monoclonal antibodies or small molecules could modulate TREM2 signaling to mitigate disease progression [189].

### 5.2 BMAL1

Brain and muscle arnt-like protein 1 (Bmal1) is a central component of the mammalian biological clock [195], essential for the regulation of circadian rhythms and the maintenance of cellular and organ physiological functions. Bmal1 expression decreases with aging [196], significantly influencing the aging process and modulating the progression of age-related diseases, including AD and OP [197].

Dysfunction of circadian rhythm has long been associated with AD-related neurodegeneration. Experimental studies demonstrate that mice with circadian rhythm disturbances exhibit loss of dendritic length, reduced neuronal complexity in the limbic prefrontal cortex, and impaired cognitive flexibility [198]. Peripheral Bmal1 deficiency in the brain parenchyma has been shown to upregulate ApoE expression and promote deposition of fibrous plaques [199]. Interestingly, Aβ pathology may exacerbate circadian rhythm disruption by degrading BMAL1, creating a vicious cycle that accelerates neurodegeneration [200]. Moreover, deletion of BMAL1 has been linked to a protective activation state in astrocytes and a reduction in tau pathology levels in vivo [201]. Therefore, strategies aimed at restoring BMAL1 levels to induce protective activation of astrocytes early in the disease process or by counteracting Aβ-induced degradation of BMAL1 may represent promising avenues for AD prevention and treatment.

In bone biology, Bmal1 plays an essential role in regulating mesenchymal cell differentiation and bone metabolism. Bmal1 deficiency inhibits osteoblast and chondrocyte differentiation, while promoting osteoclast differentiation and bone resorption [202]. Bmal1 is also involved in rhythmic oscillations of bone formation, where it promotes osteogenesis and inhibits adipogenesis. Bmal1 enhances the differentiation of BMSCs and increases the number of functional osteoblasts through the Wnt signaling pathway while suppressing adipocyte differentiation. Furthermore, Bmal1 supports osteoblast maturation and mineralization during the later stages of bone formation [203]. Recent studies have found that overexpression of Bmal1 enhances the osteogenic capacity of BMSC [204]. This highlights its therapeutic potential for the treatment of bone loss and suggests that Bmal1 could be a novel target for the treatment of bone diseases.

The dual roles of Bmal1 in circadian regulation, AD pathogenesis, and bone metabolism underscore its importance as a potential therapeutic target. Restoring Bmal1 expression may provide benefits for both neurodegenerative and skeletal disorders. Continued exploration of its mechanisms in aging and disease progression is essential for developing targeted interventions aimed at improving cognitive and skeletal health.

### 5.3 PYK 2

Proline-rich tyrosine kinase 2 (PYK2) is an adhesion patch kinase localized to postsynaptic sites in the brain and expressed abundantly both in the CNS and hematopoietic system. In neurons, PYK2 plays a critical role as a mediator of Aβ toxicity, interacting with Fyn kinase to link Aβ effects to aberrant phosphorylation of Tau [205]. PYK2 can influence both extracellular amyloid plaque and intracellular NFT formation. Specifically, PYK2 directly phosphorylates tau and is an important component of NFT, thereby enhancing tau pathology [206]. These findings highlight the potential of PYK2 as a molecular target in mitigating the downstream effects of Aβ toxicity and tau-mediated neurodegeneration in AD. In addition to its role in CNS, PYK2 is a critical regulator of bone metabolism, particularly in osteoclast and osteoblast function. PYK2 is one of the few genes known to regulate adult bone mass predominantly through its effects on bone formation [207]. PYK2 mediates osteoclast-bone interactions by serving as a major adhesion-inducing tyrosine kinase in osteoclasts. The adhesion of PYK2 to bone matrix induces osteoclast differentiation [208]. In vitro studies further confirm that PYK2 plays an active role in osteoclast maturation and bone resorption [207]. Interestingly, PYK2 deficiency has been found to promote bone formation and enhance the effects of estrogen on the formation and mineralization of the OB matrix, suggesting a dual regulatory role for PYK2 in both osteoclast and osteoblast activity [209]. Recent studies have also identified novel therapeutic approaches targeting PYK2-related pathways. For example, puerarin has been shown to inhibit the integrin-β3-mediated Pyk2-Src-Cbl pathway, thereby interfering with f-actin formation in osteoclasts and suppressing bone resorption [210]. These findings provide promising new perspectives for the treatment of OP.

### 5.4 MARK3

Microtubule affinity-regulated kinase 3 (MARK3) is a conserved serine/threonine kinase that regulates cell polarity and the cell cycle. MARK3 has been identified in a subset of the granulovacuolar neurodegeneration (GVD) -containing neurons and is strongly associated with the phosphorylation of tau at Ser 262 [211]. This suggests that MARK3 contributes to early tau phosphorylation, a hallmark of AD.

In bone metabolism, MARK3 significantly influences bone mass by disrupting cellular signals in osteoblasts. Both human and mouse studies have revealed that MARK3 transcript levels are negatively correlated with BMD [212]. Functional assessment in mice has shown that loss of function of MARK3, whether globally or specifically in osteoblasts, results in increased bone mass. MARK3-deficient osteoblasts exhibit enhanced matrix mineralization compared to controls, indicating that MARK3 negatively regulates osteoblast activity [213]. Additionally, alterations in MARK3 expression disrupt osteoblast signaling and affect osteoclast differentiation and bone mass [214] (Table 3).

**Table 3:** Potential shared genetic factors between AD and OP

Genes	Association mechanism	Refs
**TREM2**	Regulates bone resorption and neuroinflammation	[34, 35]
**BMAL1**	Regulates circadian rhythm, bone formation, and cognitive function	[36, 37]
**PYK 2**	Affects the formation of amyloid plaques and NFTs, and affects bone formation	[38, 39]
**MARK3**	Affects early tau phosphorylation in AD; Disrupts osteoblast signaling transduction	[40, 41]
**APOE**	Affects the clearance of Aβ; Promotes OB differentiation and inhibits OC formation	[42-44]
**VPS35**	Affects neuronal function and bone formation	[45-47]
**DKK1**	Regulates tau phosphorylation and bone homeostasis through the Wnt signaling pathway	[48-50]

AD, Alzheimer's disease; OP, osteoporosis; NFTs, neurofibrillary tangles; Aβ, amyloid beta; OB, Osteoblast; OC, osteoclas

## 6. Potential common signaling pathways

### 6.1 Wnt/β-catenin signaling pathway

An increasing amount of research underscores the critical role of the Wnt/β-catenin signaling pathway in AD and OP. Dysregulation of this pathway is closely linked to Aβ formation, Tau protein phosphorylation, and synaptic dysfunction [225]. Synapse loss is a prominent early feature of AD and is closely associated with cognitive deficits in AD [226]. The Wnt/β-Catenin signaling pathway promotes neuronal survival and neurogenesis and enhances synaptic plasticity. Recent studies have found that Aβ oligomers directly inhibit the Wnt/β-catenin signaling in endothelial cells, leading to BBB dysfunction and further accelerating AD progression [227].

In the skeletal system, the Wnt/β-catenin pathway is a crucial regulator of BMSC proliferation and differentiation into osteoblasts [228]. β-catenin plays a pivotal role in osteoblast proliferation, differentiation, and activity. Degradation of β-catenin results in impaired osteoblast function, increased serum ALP activity, and reduced BMD, all contributing to OP [229]. Genetic studies have further highlighted the role of this pathway in bone health. For instance, mutations in LRP5, a gene associated with Wnt/β-catenin signaling, have been linked to an increased incidence of osteoporosis or osteomalacia [230].

Animal studies also show that activation of the Wnt/β-catenin signaling pathway significantly increased osteoblast activation and bone matrix mineralization in ovariectomized mouse models [231]. The Wnt signaling pathway has led to the development of romosozumab for OP treatment [232]. Additionally, DKK1, a key antagonist of the Wnt signaling pathway, links brain and bone health and may serve as a therapeutic target for AD and OP [232-236].

### 6.2 TGF-β signaling pathway

Transforming growth factor beta (TGF-β), a multifunctional cytokine belonging to the transforming growth factor superfamily, has an important regulatory role in cell growth, differentiation, and immune function. Humans have three TGF-β isoforms: TGF-β1, TGF-β2 and TGF-β3. Aging is associated with a suppression of TGF-β1 expression and inhibition of the TGF-β/Smad signaling pathway, which leads to cytotoxic activation of microglia and mediates neurodegeneration [232]. These changes suggest that age-related declines in TGF-β signaling may be an early indicator of AD.

TGF-β is involved in multiple pathways influencing AD pathogenesis. For instance, the microglia APOE4-ITGB8-TGF-β pathway has been identified as a negative regulator of microglia's pathological response to AD [233]. Additionally, TGF-β1 exerts neuroprotective effects by activating the PI3K/Akt/Wnt/β-catenin signaling pathway [234]. However, disruption of the BBB triggers hyperactivation of the TGF-β signaling pathway in astrocytes, contributing to cognitive deficits in both animal models and humans [235].

In bone biology, TGF-β plays a dual regulatory role in the activity of osteoblasts and osteoclasts [236], essential for embryonic skeletal development and postnatal bone homeostasis [237]. During bone remodeling, TGF-β can promote the proliferation and early differentiation of bone progenitor cells via the MAPK-Smad2/3 pathway [238]. TGF-β exhibits both promotive and inhibitory effects on osteoclasts and is involved in bone extracellular matrix metabolism and chondrocyte production [239]. These findings indicate that the TGF-β signaling pathway influences both AD pathology and bone metabolism by crosstalking with other pathways, such as Wnt/β-catenin and MAPK.

### 6.3 NF-κB signaling pathway

NF-κB is a transcription factor central to the regulation of neuroinflammation and glial activation in the CNS. NF-κB can be activated by Aβ, which further promotes the production of Aβ [240], creating a vicious cycle that accelerates AD progression. Tau proteins also activate the NF-κB signaling pathway, driving microglia into an inflammatory state [241]. Inhibition of the microglia NF-κB signaling pathway in a tau protein-triggered mouse model of AD has been shown to reverse cognitive deficits, restoring the learning and memory abilities of the animals [241]. Additionally, microRNA-22-3p has been identified as a potential therapeutic agent for AD [242] by acting on Sox9 through the NF-κB signaling pathway. Given its central role in neuroinflammation and its regulation of senescence, NF-κB is a promising target for the treatment of AD.

The NF-κB signaling pathway also plays a pivotal role in regulating the differentiation and proliferation of osteoblasts and osteoclasts. Activation of the NF-κB pathway inhibits osteoblast proliferation and differentiation, while its blockade reduces inflammation-induced damage to osteoblasts [243]. The RANKL/RANK/OPG axis is a key regulator of osteoclastogenesis and bone resorption, operating through the NF-κB signaling pathway [244].RANKL binding to its receptor RANK activates NF-κB, as well as three mitogen-activated protein kinase (MAPK) pathways, which subsequently NF-κB interact with nuclear factor of activated T-cells cytoplasmic 1 (NFATc1) and c-fos transcription factors to drive osteoclast formation [245]. In contrast, osteoprotectin (OPG), a decoy receptor for RANKL, inhibits RANK signaling and protects against bone resorption [246]. Mice lacking OPG exhibit increased osteoclast activity, leading to severe bone loss and OP [247].

**Figure 2.**
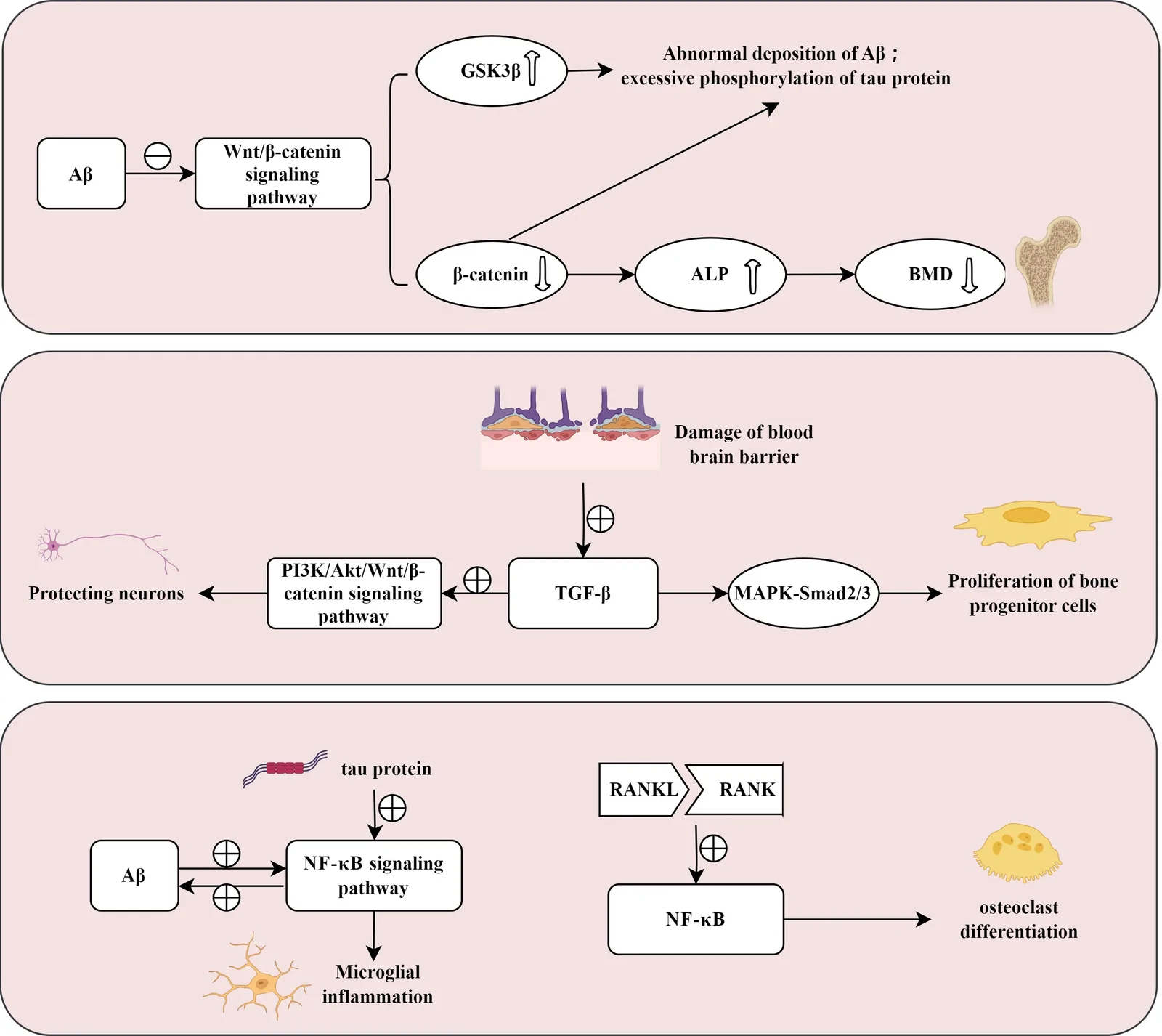
**Potential shared signaling pathways between AD and OP.** AD, Alzheimer's disease; OP, osteoporosis; Aβ, amyloid beta; BMD, bone mineral density; ALP, Alkaline phosphatase; RANK, the receptor activator of nuclear factor kappa B; RANKL, receptor activator of NF-kappaB ligand (Created with BioRender.com).

From a therapeutic perspective, the RANKL/RANK/OPG pathway is an important target for anti-OP drug development. Denosumab, a monoclonal antibody against RANKL, has been approved for the treatment of postmenopausal OP, male OP, and GIOP [248]. These findings underline the critical role of the NF-κB signaling pathway in bone metabolism and its potential as a therapeutic target for OP and AD (Fig 2).

## 7. Effects of Bone-derived proteins and extracellular vesicles (EVs) on AD

EVs are a collective term for membrane-bound vesicles released by cells into the extracellular environment, serving as carriers of biologically active molecules such as proteins, lipids, and nucleic acids. Based on their size and release mechanism, EVs are classified into three types: apoptotic vesicles (1000-5000 nm), microvesicles (100-1000 nm), and exosomes (30-100 nm) [249]. EVs are widely distributed in cell culture supernatants as well as in various bodily fluids, including blood, saliva, and urine, and play a crucial role in intercellular communication, enabling long-distance transfer of biomolecules via body fluids [250].

Bone-derived EVs, including osteocyte-derived Evs (OCY-Evs) and BMSC-derived EVs (BMSC-EVs), contribute to the crosstalk between bone and brain, particularly in AD and OP [251]. These EVs can cross the BBB and participate in Aβ metabolism. For instance, BMSC-EVs have been shown to reduce Aβ plaque deposition in the cortex and hippocampus, demonstrating potential therapeutic effects in the early stages of AD [252]. Additionally, BMSC-EVs improve cognitive function in AD rats by activating the miR-29c-3p/BACE1 axis and Wnt/β-catenin pathway activation [253].

Notably, younger OCY-EVs exhibit a greater ability to cross the BBB compared to older OCY-EVs, highlighting the age-dependent decline in the protective effect of OCY-EVs against AD [254]. Moreover, intranasal (IN) delivery of MSC-EVs in 3xTg mouse models has been shown to modulate microglia phenotypes safely and effectively, suggesting a minimally invasive therapeutic route for AD [255]. However, despite these promising findings, most studies using EV-based therapies for AD remain preclinical, necessitating further exploration for clinical translation.

Bone-derived proteins transported via EVs also play important roles in AD pathogenesis. Sclerostin, secreted by osteocytes, inhibits the Wnt/β-catenin pathway in neurons, thereby increasing the expression of BACE1 and accelerating Aβ deposition [256]. Elevated serum sclerostin levels have been identified as a potential risk factor for cognitive decline [256]. Conversely, osteocalcin (OCN), another bone-derived protein, reduces Aβ deposition in the hippocampus and cortex and improves cognitive dysfunction in AD mouse models [257]. These findings suggest that targeting bone-derived proteins and EVs could provide novel therapeutic strategies for AD by modulating the bone-brain axis.

## 8. Effects of brain-derived proteins and EVs on bone homeostasis

Brain-derived proteins and EVs also play critical roles in bone metabolism, further highlighting the bidirectional nature of the bone-brain axis. Brain-derived neurotrophic factor (BDNF (, a key member of the neurotrophic factors family, has been implicated in both cognitive function and bone formation. Intranasal administration of miR-206-3p antagomir-loaded MSC-EVs has been shown to target the brain, upregulate the level of BDNF, reduce Aβ deposition, and improve learning and memory in AD model mice [258]. BDNF and its receptor TrkB are expressed at various stages of bone formation and are upregulated in human osteoblasts. BDNF contributes to osteogenesis, promotes fracture healing [259], and stimulates cell differentiation for new bone formation [260].

Another brain-derived factor, cellular communication network factor 3 (CCN3), secreted by arcuate nucleus KISS1 neurons (ARC^KISS1^) in the maternal brain, has been shown to promote osteogenesis, bone formation, and fracture repair [261]. Similarly, brain-derived EVs influence bone metabolism by transporting microRNAs (miRNAs) that regulate osteogenic differentiation. For example, miRNA-483-5p in brain-derived EVs inhibits osteogenic differentiation and promotes adipogenesis of BMSCs, contributing to bone loss in AD [262]. On the other hand, plasma-derived small EVs (sEVs) from damaged neurons carrying microRNA-328a-3p and miR-150-5p have been shown to promote osteogenesis and bone repair by targeting osteogenic progenitor cells [263]. The application of MSC-EVs in OP therapy is still in its early stages [264]. Further clinical trials are needed to advance non-coding RNA-based therapeutics utilizing EVs as drug delivery systems for OP.

**Figure 3.**
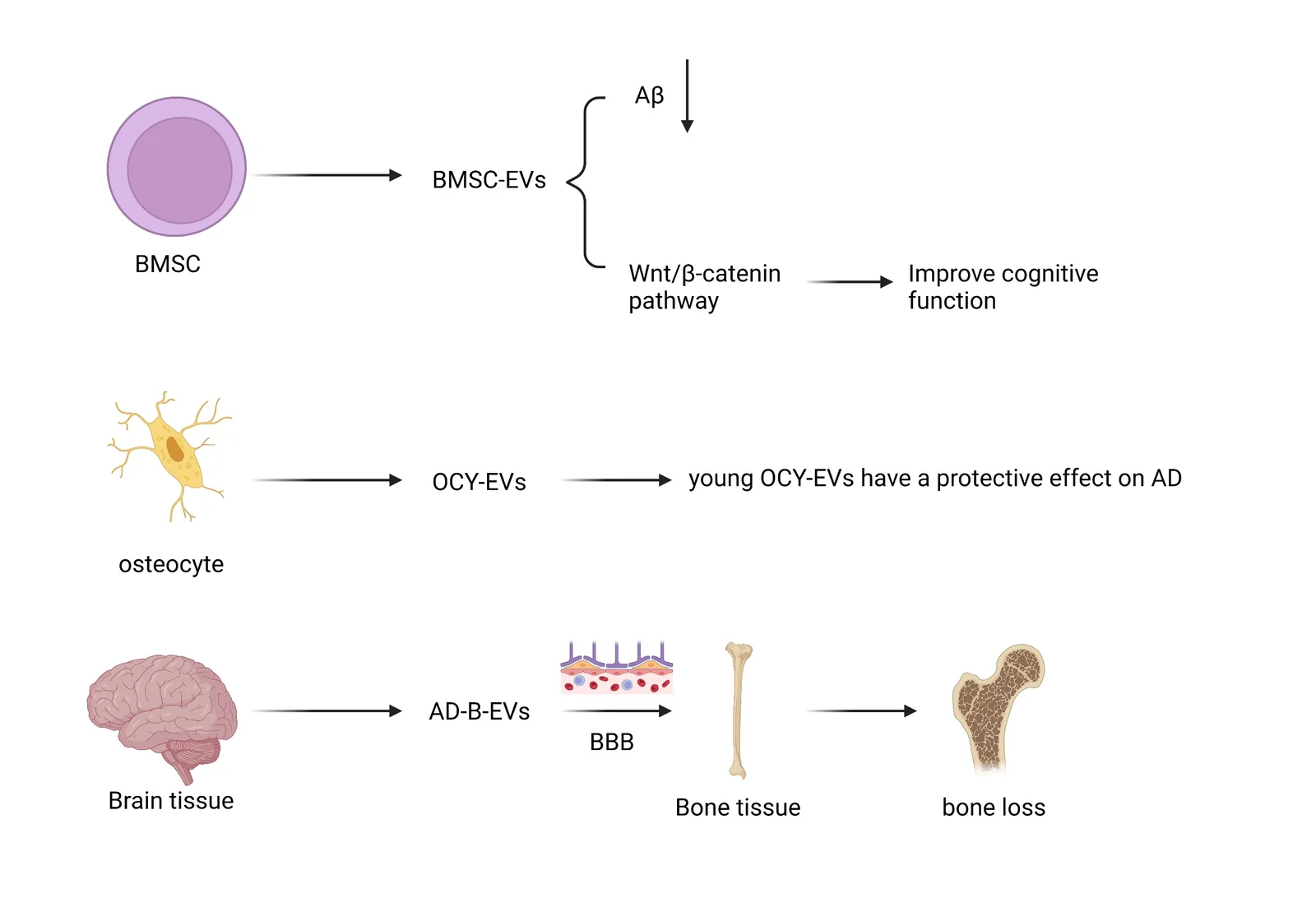
**The effect of extracellular vesicles on AD and OP**. AD, Alzheimer's disease; OP, osteoporosis; BMSC, bone marrow mesenchymal stem cell; BMSC-EVs, bone marrow mesenchymal stem cell-derived EVs; Aβ, amyloid beta; OCY-EVs, osteocyte-derived Evs; AD-B-EVs, AD brain-derived EVs; BBB,the blood-brain barrier (Created with BioRender.com)

EVs act as critical mediators of communication between the brain and bone, supporting cognitive function and maintaining bone homeostasis within the bone-brain axis. However, while preclinical studies offer promising insights into the therapeutic potential of EVs in both AD and OP, their clinical applications remain to be fully explored. Future research targeting EV-mediated pathways and bone-derived proteins may open new avenues for treating these interconnected diseases (Fig 3).

## 9. Practical and clinical implications

The current treatments for AD and OP are primarily limited to symptomatic relief, with no curative therapies available. The complexity of AD and OP comorbidity highlights the necessity of developing novel therapeutic approaches that not only target neurodegeneration but also address bone loss and dysregulated bone metabolism in AD patients. Targeting AD-related pathways may provide new avenues for OP treatment, while targeting OP-related pathways could provide insights into AD therapies. However, the efficacy and safety of anti-osteoporotic drugs in AD patients and anti-AD drugs in OP patients remain largely unexplored, underscoring the urgent need for research focused on managing neurodegeneration and bone loss in this population.

Preventive strategies targeting modifiable risk factors, such as diet, exercise, sleep, smoking, and alcohol consumption, are critical for reducing the incidence of both AD and OP. The lack of systematic studies on AD and OP comorbidity highlights the need for new animal models and well-designed clinical trials to evaluate the efficacy and safety of anti-osteoporotic drugs in AD patients.

## 10. Summary

The interplay between genetic and environmental factors plays a crucial role in developing complex diseases such as AD and OP. AD and OP have multifaceted etiologies involving multiple susceptibility genes and diverse environmental factors. Environmental factors often exacerbate the risk of AD and OP by promoting stress hormone signaling, oxidative stress, and chronic inflammation. This review summarizes the current understanding of how environmental factors influence the onset and progression of AD and OP and highlights the genetic mechanisms underlying these conditions. However, the intricate interactions between environmental and genetic predispositions remain insufficiently explored. Notably, gene-gene and gene-environment interactions may also vary significantly across populations, particularly in polygenic diseases like AD and OP.

The correlation between AD and OP remains incompletely understood, and the shared factors and mechanisms driving their co-pathogenesis require further investigation. Although several common genetic factors contributing to both AD and OP have been identified, additional research is needed to elucidate the interconnections between the two diseases. Future studies should investigate how specific environmental factors (e.g., metal ions and unhealthy lifestyles) interact with genetic backgrounds to influence the development and progression of AD and OP. The shared pathologic features of AD and OP present an opportunity for developing common therapeutic strategies. For example, drugs targeting the Wnt/β-catenin or TGF-βsignaling pathways could potentially enhance cognitive function and BMD. Additionally, future research should prioritize the exploration of novel cell-based therapeutic approaches, particularly those involving bone-secreted proteins and brain-derived proteins, given the intricate interactions between bone and brain.

Understanding the complex link between AD and OP holds promise not only for improving early diagnosis and treatment but also for discovering innovative therapeutic strategies that benefit both diseases. Furthermore, the application of advanced techniques such as big data and artificial intelligence could aid in identifying novel biomarkers and developing targeted treatments for AD and OP. This integration of interdisciplinary approaches is critical for bridging existing knowledge gaps and advancing therapeutic development.

Future studies should integrate GWAS data with environmental data and apply machine learning methods to predict the risk of AD and OP under specific environmental exposures. Additionally, integrating genomics, transcriptomics, proteomics, and metabolomics could help identify shared molecular pathways and mechanisms underlying AD and OP. Advanced techniques such as single-cell sequencing and spatial transcriptomics could refine our understanding of pathological changes at the cellular level. The development of dual-purpose drugs and therapeutic strategies that target both AD and OP should be pursued, with their efficacy and safety rigorously tested in clinical trials.
